# Cost-effectiveness of a lifestyle intervention for people with a serious mental illness (SMILE): design of a pragmatic cluster-randomised controlled trial

**DOI:** 10.1186/s12888-019-2132-5

**Published:** 2019-05-16

**Authors:** Florine S. Walburg, Berno van Meijel, Maurits W. van Tulder, Marcel C. Adriaanse

**Affiliations:** 1 Department of Health Sciences and Amsterdam Public Health Research Institute, Faculty of Science, Vrije Universiteit, Amsterdam, The Netherlands; 2 Department of Psychiatry, UMC Amsterdam and Amsterdam Public Health research institute, Amsterdam, The Netherlands; 3 Department of Health, Sports & Welfare, Cluster Nursing, Inholland University of Applied Sciences, Amsterdam, The Netherlands; 4 Parnassia Psychiatric Institute, The Hague, The Netherlands; 5 GGZ-VS Academy for Masters in Advanced Nursing Practice, Utrecht, The Netherlands; 6 GGZ inGeest Specialized Mental Health Care, Amsterdam, The Netherlands

**Keywords:** Lifestyle, Intervention, Serious mental illness, Cost-effectiveness, Weight loss, Cardiovascular risks, Physical health, FACT

## Abstract

**Background:**

Cardiovascular disease is the leading cause of the estimated 11–25 years reduced life expectancy for persons with serious mental illness (SMI). This excess cardiovascular mortality is primarily attributable to obesity, diabetes, hypertension, and dyslipidaemia. Obesity is associated with a sedentary lifestyle, limited physical activity and an unhealthy diet. Lifestyle interventions for persons with SMI seem promising in reducing weight and cardiovascular risk. The aim of this study is to evaluate the effectiveness and cost-effectiveness of a lifestyle intervention among persons with SMI in an outpatient treatment setting.

**Methods:**

The Serious Mental Illness Lifestyle Evaluation (SMILE) study is a cluster-randomized controlled trial including an economic evaluation in approximately 18 Flexible Assertive Community Treatment (FACT) teams in the Netherlands. The intervention aims at a healthy diet and increased physical activity. Randomisation takes place at the level of participating FACT-teams. We aim to include 260 outpatients with SMI and a body mass index of 27 or higher who will either receive the lifestyle intervention or usual care. The intervention will last 12 months and consists of weekly 2-h group meetings delivered over the first 6 months. The next 6 months will include monthly group meetings, supplemented with regular individual contacts. Primary outcome is weight loss. Secondary outcomes are metabolic parameters (waist circumference, lipids, blood pressure, glucose), quality of life and health related self-efficacy. Costs will be measured from a societal perspective and include costs of the lifestyle program, health care utilization, medication and lost productivity. Measurements will be performed at baseline and 3, 6 and 12 months.

**Discussion:**

The SMILE intervention for persons with SMI will provide important information on the effectiveness, cost-effectiveness, feasibility and delivery of a group-based lifestyle intervention in a Dutch outpatient treatment setting.

**Trial registration:**

Dutch Trial Registration NL6660, registration date: 16 November 2017.

## Background

People with a serious mental illness (SMI) have an estimated reduced life expectancy of 11–25 years in comparison to the general population [1–3] This reduced life expectancy is primarily caused by cardiovascular disease [4]. The high risk of cardiovascular disease within this population is mainly due to unhealthy lifestyle behaviour, in combination with the use of antipsychotic medication. People with SMI are more likely to suffer from obesity, hypertension, diabetes and dyslipidaemia [4, 5]. Obesity in particular has been associated with limited physical activity, an unhealthy diet and medication side effects in people with SMI [4]. People with SMI also have less access to healthcare and receive insufficient monitoring and therapy for cardiovascular risks [4–8].

In 2014, the NICE U.K. guidelines of prevention and management of psychosis and schizophrenia in adults were updated [9]. These guidelines recommend that cardiovascular risks should be regularly monitored and healthy eating and physical activity programs should be offered to people taking antipsychotics [9]. In 2015, the multidisciplinary Dutch guidelines for lifestyle and somatic screening in persons with SMI were published to stress the importance of physical health and a healthy lifestyle in persons with SMI [10]. These guidelines likewise mention the significance of lifestyle interventions in daily care in order to reduce somatic health problems in this population.

Even though some studies show mixed results, research indicates that lifestyle interventions might be effective in stimulating healthier lifestyle in people with SMI [11–13]. The STRIDE study - conducted in a community setting in the U.S. - examined a 12-month lifestyle program designed to promote weight loss and reduce cardiovascular risks in people with SMI taking anti-psychotic medication through a healthy diet and increased physical activity [14]. The STRIDE program improved weight, fasting glucose and health-related self-efficacy [15, 16].

It is possible that mental health care workers currently lack sufficient knowledge, techniques and resources to address healthy lifestyle change in order to follow these guidelines in busy and demanding daily practice. Therefore, health care practice needs an innovative approach that is practically feasible for mental health care workers and effectively stimulates healthy lifestyle behaviours among people with SMI.

In the Netherlands, the majority of people with SMI who are living at home receive treatment by Flexible Assertive Community Treatment (FACT) teams. Care from FACT-teams includes illness management, symptom treatment, guidance and practical assistance in daily living, rehabilitation and recovery support [17, 18]. There is little research available regarding an effective lifestyle intervention implemented in Dutch psychiatric ambulatory service. It is unknown whether a lifestyle intervention would be feasible, effective and cost-effective within FACT-team regular care and could effectively contribute to lifestyle changes in this population. Therefore, this study will aim to evaluate the effectiveness and cost-effectiveness of a lifestyle intervention in persons with SMI in Dutch FACT-teams in comparison to usual care; the SMILE (Serious Mental Illness Lifestyle Evaluation) study.

## Methods

### Study design

We will perform a pragmatic multi-centre cluster randomized controlled trial including an economic evaluation from a societal perspective. Follow-up will be 1 year.

### Setting

We will recruit approximately 18 FACT-teams from different mental health care organisations throughout different regions in the Netherlands. To recruit these FACT-teams, we will approach several teams with brief information about the study. If a FACT-team is interested in the study, research staff will provide a presentation for all the members of the FACT-team. Based on this presentation, teams will decide if they are interested in joining the study or not. All FACT-teams will have be certified as “FACT” by the Certification Center ACT and FACT at the beginning of the study.

### Participants

The study population will consist of adult people with SMI, age ≥ 18, a body mass index ≥27, treated by FACT-teams, who in addition are able and willing to participate in the intervention and sign informed consent. A potential participant who meets any of the following criteria will be excluded from participation: contra-indications (to be assessed by the coordinating mental health professional or treating physician) for participation due to acute psychiatric crisis or somatic disease (e.g. bariatric surgery, cancer, heart attack or stroke); individuals with cognitive impairment sufficient to interfere with their ability to provide informed consent, complete study questionnaires, or participate in a group intervention; women who are pregnant, breastfeeding or planning for a pregnancy during the course of the study; participants who are not able to communicate in the Dutch language. All participants will receive 10 Euros for each of the measurements performed at baseline, 6 months and twelve months.

### Randomisation

FACT-teams will be randomly allocated to serve as intervention (where the SMILE lifestyle intervention will be given) or control FACT-teams (where care as usual will be continued). Cluster-randomization will be applied in order to avoid contamination between the intervention and control groups. Randomisation will be performed on the level of mental health care centres to optimize the comparability between FACT-teams in order to reduce bias. Randomisation will be performed by a statistician otherwise not involved in the study. All measurement assessments will be performed by trained research assistants and trained mental healthcare staff from the corresponding mental health care centre. They will use the same standardised protocols for all measurements. Blinding of health care workers from FACT-teams and participants is not possible due to the nature of the intervention.

### Recruitment

**Fig. 1:**
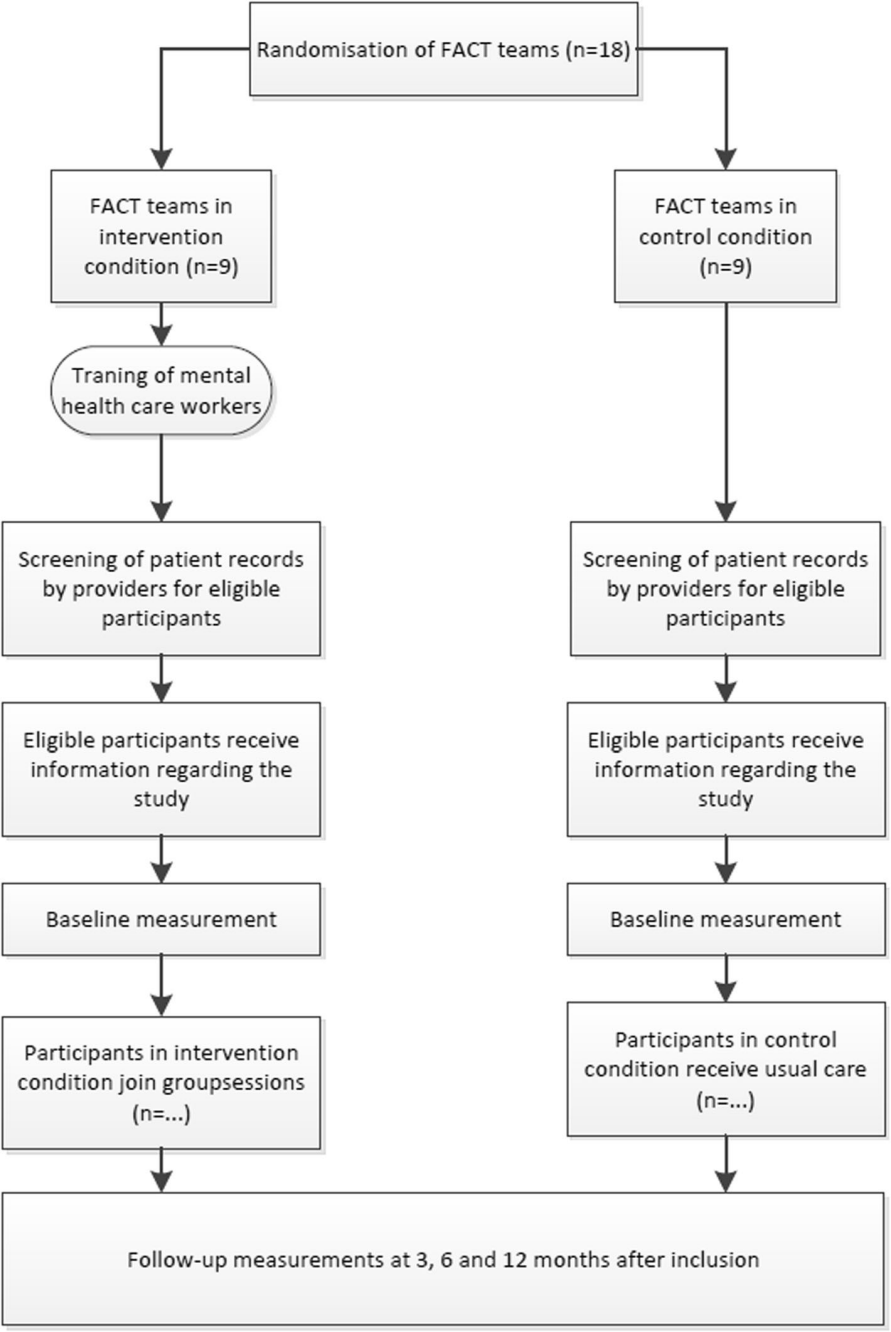
Overview of the study design and participant flow

To recruit eligible participants, all FACT-teams make a list of potentially eligible participants. The list will be assessed by the coordinating mental health professional for any contra-indications. Potential participants will be approached by the mental healthcare staff from the corresponding FACT-team with information about the study. Before the first measurements take place, the participant will be asked to sign informed consent. An overview of the study design and patient flow is presented in Fig. 1.

### Intervention

The SMILE intervention is primarily modelled after the STRIDE intervention developed by Green et al. [15]. The STRIDE lifestyle intervention is modified for persons with SMI and is based on the PREMIER lifestyle intervention with the DASH (Dietary Approaches to Stop Hypertension) dietary pattern (18–24), both developed for people without mental illnesses. It incorporates behaviour change theories such as the trans theoretical model [19, 20] and motivational theory [21–23]. The SMILE intervention primarily aims at a healthy diet and increased physical activity. The intervention will be given by trained mental health care workers.

The intervention will last 12 months and consists of an initial intervention (first 6 months) and a maintenance phase (second 6 months).

#### Initial intervention

During the first phase, participants will follow weekly 2-h group sessions during the first 6 months. In the group sessions major focus is on topics such as nutrition, increased (moderate) physical activity and lifestyle changes. Topics such as sleep, stress, effects of mental health on behavioural life-style changes, effect of medication use on weight gain, influence of the social environment on life-style behaviour, self-monitoring and goal setting concerning life-style activities are integrated within the program. In each session, participants make their own personal goals, thus tailoring the intervention to the needs, possibilities and motivation of the participant. Contacts will be collaborative, discussing lifestyle change efforts, with guided problem solving. Thirty minutes of the 2-h group sessions will be spent on a physical exercise activity. For each session group leaders will use the SMILE protocol handbook, which describes the content of the intervention.

#### Maintenance phase

The maintenance phase includes 6 months of group sessions, with monthly 2-h group sessions focusing on maintaining weight loss. Sessions will be supplemented with monthly individual telephone sessions and/or email contact with group leaders.

#### Adherence

Intervention group sessions not attended (no-shows) will be assessed by the staff of the according FACT-team. Researchers will visit group sessions of all participating teams in order to improve adherence to the intervention.

### Usual care

Mental health care workers from FACT-teams in the control group will not receive any training. They will provide care as usual.

### Training of mental health care workers

The mental health care workers from the FACT-teams randomised to the intervention group will follow a two-day training. At least two mental health workers of each team will receive the training. They will function as role models for their colleagues within the FACT-team on the subject of lifestyle for persons with SMI and will be the group leaders of the group sessions. The training will consist of: (a) consequences of an unhealthy lifestyle including determinants of increased weight and cardiovascular risks for persons with SMI and medication side effects, (b) elements of motivational interviewing and (c) components of the lifestyle intervention (including contents of group sessions, nutrition components and physical activity guidelines), and (d) a workshop in which different group sessions are rehearsed and reviewed. In addition, attention is given on how to inform and motivate potential participants in joining the lifestyle intervention. The training will be set up and given by two highly experienced trainers with ample expertise on the subject of lifestyle within mental health care and more specifically in FACT-teams. FACT-teams can decide which health care workers will be trained for the intervention. This is done to promote a bigger resemblance to general care and enhance the generalisability of the study.

#### Demographics

Demographic data (age, gender, ethnicity, marital status, education level, employment status, diagnosis of SMI, number of years receiving mental care in FACT-team) will be collected directly from the patient records or patient reports.

### Main study outcomes

#### Primary outcome

The primary outcome is weight loss (kg). Body weight will be measured with participants wearing light clothing (i.e. with empty pockets, no belt) and no shoes or jackets to the nearest 0.1 kg. All assessors of each FACT-team will use the same provided digital scale (Beurer GS210).

#### Clinical outcomes

Secondary clinical outcomes include body mass index (kg/m2), waist circumference (cm), lipid profiles (LDL, HDL, total cholesterol, triglycerides), glucose metabolism (serum and fasting glucose), and systolic and diastolic blood pressure. All clinical outcomes will be measured by trained mental health professional staff or trained research staff.

#### Patient reports

Quality of life will be measured using the Dutch version of the Short Form-12 (SF-12) and the EuroQol (EQ-5D-5 L). The SF-12 is a generic, reliable and validated instrument, containing 12 items derived from the Short Form-36 questionnaire [24]. The physical and mental component summary scores of the SF-12 will be used. Dutch age- and sex-standardized population norms are available [25]. Additionally, the EQ-5D-5 L scores will be used to compute quality adjusted life years (QALYs) and valuing health [26]. The EQ-5D-5 L rates self-care, mobility, pain, psychological functioning (anxiety/ depression), and usual activities on a 5-point scale. The EQ-5D-5 L health states will be converted into utility scores using the Dutch tariff [27]. QALYs will be calculated using linear interpolation between measurement points. Using the area under the curve method, QALYs were calculated by multiplying the amount of time a patient spent in a specific health state with the utility score associated with that health state. Transitions between health states will be linearly interpolated. Smoking status is based on self-report; patients will be asked if they are current smokers and, if so, how many cigarettes they smoked in the last 7 days. Additionally, at 6 months and 12 months follow-up measurements patients who mentioned to be current smokers will be asked if they want to be referred to a smoking cessation program or another kind of help for smoking cessation (such as guidance by a caregiver or physician).

Health-related self-efficacy is measured by the Patient Activation Measure (PAM-13), a reliable questionnaire which contains 13 items derived from the original PAM-22 [28]. The questionnaire assesses the participants’ self-reported knowledge, skills and confidence for health-related self-efficacy. Satisfaction and personal view with respect to physical and mental health, nutrition status, physical activity status and sleep will be asked by giving a score from zero to ten concerning the past 2 weeks.

All questionnaires will be completed on paper or digitally (if participant is in the possession of a smartphone or internet). In case participants experience concentration or cognitive problems when filling in the questionnaires, participants will be helped by their case workers or assessors to support them. Staff and assessors will be instructed on how to support a participant in filling in questionnaires without influencing the answers.

#### Costs

For the economic evaluation a short questionnaire will be used based on the original TiC-P questionnaire (30). The questionnaire includes questions regarding health care utilization, work absenteeism and presenteeism. Additionally, information regarding medication use is derived from the pharmacy of the participant. The costs of the lifestyle program will be measured using a bottom-up approach.

#### Economic evaluation

Both a cost-effectiveness analysis (CEA) and a cost-utility analysis (CUA) will be performed from a societal perspective. Missing cost and effect data will be imputed using Multiple Imputation with Chained Equations (MICE) according to the MICE algorithm developed by Van Buuren [29]. In addition, incremental cost-effectiveness ratios (ICERs) will be calculated. Bias-corrected and accelerated bootstrapping with 5000 replications will be used to estimate statistical uncertainty surrounding the ICERs. Uncertainty surrounding ICERs will be graphically presented on cost-effectiveness planes.

A budget impact analysis (BIA) will be conducted from the perspective of health-care decision makers. Perspectives that will be considered are the societal and the government (Budget Kader Zorg) perspective. Different scenarios will be evaluated including the following: 1) the lifestyle intervention is not implemented, i.e., all patients receive usual care, 2) the intervention is offered to the whole patient population, 3) the intervention is implemented over a 4 years period (25% of the patient population per year), and 4) the intervention is only offered to specific subgroups of the potential patient population. One-way sensitivity analyses will be performed in which the adoption rate of the lifestyle intervention and the impact on long-term clinical outcomes will be varied.

#### Process evaluation

A process evaluation will be performed in order to identify barriers and facilitators of the SMILE program and experiences of FACT team members with the delivery of the training and intervention. This will be done by organizing focus groups and/or semi-structured interviews with both clients and trained mental health care workers. An overview of the SMILE measurements, instruments and data collection schedule is provided in Table 1.

**Table 1:** SMILE measurements, instruments and data collection schedule

	Baseline	3 month	6 month	12 month
PRIMARY				
Weight loss (kg)	x	X	x	x
CLINICAL OUTCOMES				
BMI (kg/m^2^)	x	X	x	x
Waist circumference (cm)	x	X	x	x
Lipid profiles*	x			x
Glucose metabolism (mmol/l)	x			x
Systolic BP (mm/hg)	x		x	x
Diastolic BP (mm/hg)	x		x	x
PATIENT REPORTS				
Quality of life (SF12 and EQ-5D-5 L)	x		x	x
Health related self-efficacy (PAM-13)	x		x	x
Self-reported views on physical activity, nutrition status and sleep	x		x	x
Smoking status	x		x	x
DEMOGRAPHICS				
Age, gender, ethnicity	x			
Education level	x			
Marital status	x			x
Employment status	x			x
Diagnosis of SMI	x			
Number of years receiving ambulatory mental care	x			
COSTS				
Health care utilization and lost productivity questionnaire	x		x	x
Medication use	x		x	x
OTHER				
Compliance to group sessions	Weekly /monthly			

*Lipid profiles include fasting cholesterol (HDL, LDL, total) and fasting triglycerides (mmol/l)

### Sample size

This trial is powered to detect a mean difference of 4 kg in weight reduction after 1 year between the intervention and usual care group [15, 30]. Using a power of 0.80 and an alpha of 0.05, two groups of 100 participants are needed to detect a difference of 4 kg. Assuming an ICC of 0.01 and a dropout rate of 20% [15, 30, 31], we aim to recruit 260 participants in total.

### Statistical analysis

Baseline demographic characteristics of the participants in the intervention and control group will be presented using descriptive statistics (mean (standard deviation), median (range) or (proportion)).

All analysis will be on an intention-to-treat basis. Multivariable analyses techniques will be used to correct for prognostic variables. To study the effectiveness of the lifestyle intervention versus usual care in reducing weight, linear mixed models will be used. We will examine between-group differences for various conceptualizations for the primary outcome weight, including percentage weight change, the proportions of people with SMI who lose at least 5 and 10% of their baseline weight. Also, linear and logistic mixed models (depending on the outcome) will be used to test the differences between both groups regarding the secondary clinical outcome parameters; waist circumference, lipids, blood pressure and glucose. Furthermore, we will examine whether there was an effect of the intervention on patient reported outcomes, including perceived health, quality of life and health related self-efficacy. For this purpose, we will also use linear mixed models. Next, we will evaluate whether dose, defined as the number of intervention sessions, is related to change in the outcomes across time among participants who receive the intervention.

Finally, in case of unequal distributions of demographic variables between the two treatment groups, multivariate analyses techniques will be used to correct for these differences.

## Discussion

Research suggest lifestyle interventions can be effective in improving physical health of people with SMI. However, there is currently no evidence of cost-effectiveness regarding a lifestyle intervention for people with SMI in a Dutch outpatient setting. Because lifestyle interventions are typically intensive and include many group and individual sessions, the costs of these programs are substantial. Given the limited healthcare resources it is of utmost importance to evaluate whether the additional costs are worth the effects. Given the large number of persons with SMI and cardiovascular risks, and the associated high burden for the patient, family members and society, this study is a matter of urgency. This paper presents the design of a cluster randomized controlled trial to evaluate the effectiveness and cost-effectiveness of a lifestyle intervention for people with SMI in the Dutch outpatient psychiatric treatment setting.

The SMILE intervention is primarily modelled after the STRIDE intervention [14], developed for persons with SMI and effective in reducing weight and cardiovascular risk factors [15]. The SMILE intervention will be carried out by trained mental health nurses working in FACT-teams in the Netherlands. It is hypothesized that the SMILE intervention is more effective than usual care in reducing weight, reducing cardiovascular risk factors, improving quality of life. Although the costs of the intervention will be higher than usual care, these costs might be outweighed by lower costs of other healthcare utilization and increased productivity. This study has substantial societal value as it provides financing organizations and governments with better insights how to spend the available resources in the most efficient way.

A strength of the current study is its pragmatic design. A large number of studies, specifically randomised controlled trials, use highly controlled settings which could greatly differ from regular care. In the current study, the intervention is implemented by mental health care workers from FACT-teams in the current regular care situation, with the same available time, priorities, and motivation. This pragmatic design will enhance the generalizability of the study results and therefore the possibilities to implement the SMILE intervention in a real-life mental health care setting.

The intervention is based on motivational interviewing techniques and the behavior change theories. Both are very important in order to stimulate behaviour change [32–34]. As participants formulate their own personal goals each week, they can tailor their goals to their personal needs, capabilities and motivation at that moment. However, as Dutch and U.S. mental health care differ to a certain extent, the effect of the adapted STRIDE intervention may differ within Dutch mental health care. We believe that even though the characteristics of the healthcare system differ between countries, the core principles of behaviour change are the same in each country and the difference in characteristics is not likely to have a major impact on the results of this study.

A few adaptations of the STRIDE interventions were made in order to implement the study according to practical possibilities of current mental health care and to fit Dutch guidelines and food customs [35, 36]. One of the adaptations was that the SMILE study will focus less on calorie counting. Reducing calories and monitoring is one of the core intervention components of STRIDE. Calorie density of food products is still an important topic within the group sessions, but the counting of calories is less emphasized. Furthermore, group leaders will not be able to evaluate nutrition, physical activity and sleep journals of the participants for each group session. We chose not to implement this in the intervention, as this would be too time consuming for group leaders. Such evaluations will be integrated within the “check-in” part of the 2-h group session, where progress is discussed and evaluated.

Overall, the SMILE intervention for persons with SMI will provide important and relevant information on the effectiveness, cost-effectiveness, feasibility and delivery of a lifestyle intervention in a Dutch outpatient treatment setting. The first results of this study are expected in 2020.

## Abbreviations

BMI: body mass index
CEA: Cost-Effectiveness
FACT: Flexible Assertive Community Treatment
ICERs: Incremental Cost-Effectiveness ratios
PAM 13: patient activation measure 13
SF12: Short form 12
SMI: serious mental illness
SMILE: Serious Mental Illness Evaluation Lifestyle Evaluation
